# Serotonin-Norepinephrine Reuptake Inhibitors for Pain Control: Premise and Promise

**DOI:** 10.2174/157015909790031201

**Published:** 2009-12

**Authors:** David M Marks, Manan J Shah, Ashwin A Patkar, Prakash S Masand, Geun-Young Park, Chi-Un Pae

**Affiliations:** 1 Department of Psychiatry and Behavioral Science, Duke University Medical Center, Durham, NC, USA; 2 Department of Rehabilitation Medicine, The Catholic University of Korea College of Medicine, Seoul, Republic of Korea; 3 Department of Psychiatry, The Catholic University of Korea College of Medicine, Seoul, South Korea

**Keywords:** Role, serotonin, norepinephrine, pain control, SNRIs, modulation, pain pathways.

## Abstract

The precise mechanisms of pain perception and transmission in the central nervous system have not been fully elucidated. However, extensive data support a role for the monoamine neurotransmitters, serotonin and norepinephrine, in the modulation of pain. Experiments with animal models of pain indicate that noradrenergic interventions, and to a lesser extent serotonergic interventions, reduce pain-related behavior. This is supported by data from clinical trials in humans in which antidepressants have been shown to reduce pain and functional impairment in central and neuropathic pain  conditions. These effects are particularly well-studied in trials with serotonin-norepinephrine reuptake inhibitors (SNRIs), which have provided a useful tool in the clinician’s arsenal, particularly considering the limitations of other classes of pain medications such as opioids, anti-inflammatories, and anticonvulsants (i.e., limited efficacy, safety and tolerability issues). Moreover, painful physical symptoms are frequently comorbid with major psychiatric disorders such as major depressive disorder and anxiety disorders. This paper reviewed and summarized the rationale and potential role of SNRIs for the control  of pain including clinical and preclinical background. Currently evidence does not definitely support a role of the SNRIs, while limited data propose a putative promise of SNRIs in the treatment of pain related disorders including fibromyalgia and depressed patients with multiple somatic complaints. More researches are warranted to generalize currently available preliminary evidences.

## INTRODUCTION

Although a number of neurotransmitters, neuromodulators, and receptors are likely involved in modulating the ascending and descending pain pathways, serotonin and norepinephrine have been implicated as principal mediators of endogenous analgesic mechanisms in the descending pain pathways [22].

The precise mechanisms involved in the pathogenesis of persistent pain states are not fully understood, but there is growing recognition that the disinhibition and imbalance of serotonin and norepinephrine in endogenous pain inhibitory pathways may contribute to persistent pain [11]. It is known that the substances which have the most prominent anti-nociceptive effects are generally inhibitory neurotransmitters, and their action is primarily mediated by the activation of descending inhibitory pathways which inhibit pain [18]. Descending input from the cortex, hypothalamus, and amygdala and pretectal nucleus is provided to the midbrain periaqueductal gray, the rostroventral medulla, and the dorsolateral pontomesencephalic tegmentum [22]. Both the rostroventral medulla and dorsolateral pontomesencephalic tegmentum project to the spinal dorsal horn. The descending pain pathways form an endogenous pain-modulating circuit consisting of both inhibitory and facilitatory components [6, 18].

There have been numerous studies demonstrating the analgesic effect of antidepressants, providing growing evidence that antidepressants are beneficial in the treatment of so-called 'chronic pain' [30]. Data is strong regarding the pain-inhibiting effects of tricyclic antidepressants (TCA) and newer agents enhancing norepinephrine and serotonin neurotransmission. In contrast, review of studies with selective serotonin reuptake inhibitors (SSRIs) revealed inconsistent evidence of efficacy for migraine or tension headaches, diabetic neuropathy, and fibromyalgia, although several studies of SSRI treatment for mixed-chronic pain are universally positive [19].

Among contemporary antidepressants, currently available preclinical and clinical data indicate that serotonin-norepinephrine reuptake inhibitors (SNRIs) may be the most promising agents for the modulation of pain symptoms.

## PRECLINICAL STUDIES

The CNS pathway responsible for inhibition of pain sensation includes projections from various brainstem nuclei to the spinal cord dorsal horn *via* the dorsolateral funiculus (DLF). More specifically, DLF fibers are comprised of serotonergic projections from the raphe nuclei, dopaminergic projections from the ventral tegmental area (VTA), and noradrenergic projections from the locus coeruleus. These descending fibers suppress pain transmission at the nociceptive spinal cord neurons presumably by hyperpolarizing afferent sensory neurons using endogenous opioids, or serotonin and norepinephrine as principal inhibitory mediators. [9]. The pain pathways are presented in Fig. (**1**).

Persistent pain results from changes in sensitivity within both ascending and descending pain pathways in the brain and the spinal cord [17]. Neuropathic pain (e.g. diabetic neuropathy, postherpetic neuralgia) is a type of persistent pain that arises from functional changes occurring in the pain sensory system after peripheral nerve injury. Sustained or prolonged stimulation of sensory afferents due to tissue damage or peripheral nerve injury has been implicated in the initiation and maintenance of central neuroplastic changes culminating in central neuronal hyperexcitability; this may be complicated by reduced inhibition of nociceptive neurons by neurotransmitters, such as serotonin and norepinephrine in both spinal and supraspinal structures [18].

The inhibitory action of serotonin on structures of the dorsal horn may be mediated by activation of opioid-releasing interneurons. In animal models, naloxone, an opioid antagonist, attenuates the analgesic effect of intraspinal serotonin; similarly, serotonin antagonists interfere with analgesic effects of morphine infused in or near the spinal cord [25]. Studies have also shown that adrenergic receptors are pivotal in the control of pain management in animal models [15]. Subsequent formalin tests of rats treated with antidepressants and antagonists of monoamine receptors indicate that adreno-and serotonin receptors are associated with antinociception, indicating functional interactions between noradrenergic and serotonergic neurons as mechanisms of antidepressant-induced pain-control [43].

A number of animal studies have suggested an important role of noradrenergic and serotonergic neurotransmitters in the processing of pain. Experimental studies have shown that serotonin and norepinephrine agonists given intrathecally block pain-related behaviors [12,13], while other data suggests that serotonin agonists such as fenfluramine elicit pain-related behaviors by increasing neuronal release of substance P [14].

Additionally, serotonin receptor antagonists such as ondansetron given to rats intrathecally inhibited experimental pain response [15], suggesting that excitatory serotonergic descending pathways facilitate the expression of pain. It is likely that serotonin both inhibits and promotes pain perception by different physiological mechanisms, in contrast to norepinephrine which is predominately inhibitory. Additional evidence of the role of monoamines in pain modulation comes from studies of antidepressant administration in animal models of pain. In comparison of drugs inhibiting serotonin or norepinephrine reuptake inhibition (desipramine, reboxetine, fluoxetine and paroxetine), the norepinephrine reuptake inhibiting drugs desipramine and reboxetine reversed tactile allodynia at an overall magnitude equivalent to that of the anticonvulsant gabapentin. However, discrepant effects were observed with the SSRIs paroxetine and fluoxetine, indicating a more important role of norepinephrine in pain inhibition compared to serotonin [23].

One experimental study found that microinjection of paroxetine into the basolateral amygdala or cingulate cortex in mice reduced anxiety-related behavior, and microinjection into the primary somatosensory cortex significantly attenuated thermal hyperalgesia [24]. However, these findings have not been consistently replicated in subsequent preclinical and clinical studies. Medications enhancing neurotransmission of both noradrenergic and serotonergic pathways have shown antinociceptive properties in preclinical and clinical research. In particular, duloxetine, a potent SNRI significantly reduced pain behavior in rats during paradigms of persistent pain (formalin model) and neuropathic pain (L5/L6 spinal nerve ligation model), but not during a paradigm of acute nociceptive pain (tail-flick model) [18]. This effect of duloxetine was more potent than other SNRIs including venlafaxine, milnacipran, and the TCA amitriptyline. It should also be noted that in this experiment low doses of the SSRI paroxetine or the norepinephrine reuptake inhibitor (NRI) thionisoxetine alone did not show reduction of formalin model persistent pain behavior, which was observed when medications were combined [3]; this further supports the importance of both serotonergic and noradrenergic mechanisms in pain modulation and implies that medications affecting both neurotransmitters have greatest effect. In addition, a number of studies have demonstrated the strong effect of milnacipran in the treatment of pain in rat models. In one such experiment, intrathecal administration of milnacipran but not paroxetine had prominent antiallodynic effects in a neuropathic pain paradigm [28]. Milnacipran was also effective in acute treatment and prevention of repeated stress-induced hyperalgesia [37].

Morover, coadministration of milnacipran with tramadol potentiated the antihyperalgesic effect of tramadol in rats, indicating that milnacipran has an antihyperalgesic effect mediated by opioidergic mechanisms as well as *via* serotonergic and noradrenergic pathways [29].

In summary, preclinical data suggests that persistent and neuropathic pain may be inhibited by enhancement of norepinephrine and serotonin transmission (both neurotransmitters > norepinephrine alone > serotonin alone), and that deficiencies in one or both of these neurotransmitter systems may contribute to hyperactive pain processing.

## AFFINITY TO MONOAMINE TRANSPORTERS AND MONOAMINE REUPTAKE INHIBITION OF CURRENT SNRIS

TCAs such as imipramine and amitriptyline inhibit both serotonin and norepinephrine uptake *in vitro* to variable degrees, indicating their potential being serotonin-norepinephrine dual uptake inhibitors [5]. However, *in vivo* imipramine and amitriptyline are rapidly metabolized to secondary amines that are potent and selective NRIs for which they substantially lose the practical effects of SNRIs [5]. In addition, TCAs are notorious their inherent side effects stem by inhibition of multiple receptors such as muscarinic, α-adrenergic and histamine H_1_ receptors. Hence relatively genuine SNRIs are venlafaxine, milnacipran and duloxetine in the current market. Given a pivotal role of serotonin and norepinephrine dual reuptake inhibition for pain control, the binding affinity of SNRIs to serotonin and norepinephrine transporter and reuptake inhibition effect in the synaptic cleft may be crucial in their clinical efficacy. However, differential effect of such medications on serotonin and norepinephrine neurotransmission has been suggested. A recent study has compared the ability between duloxetine and venlafaxine to block serotonin and norepinephrine transporters *in vitro* and *in vivo* [5]. Duloxetine potently inhibits binding to the human serotonin transporters and norepinephrine transporter approximately by 100 times and 300 times greater potency, respectively, comparing with venlafaxine [5]. In addition, duloxetine inhibited serotonin and norepinephrine reuptake with K_i_ values of 4.6, 16 and 369 nM, respectively, while venlafaxine inhibited reuptake with 17 and 34-fold lower potency, respectively, comparing with duloxetine [5]. This differential biding affinity and reuptake inhibition on monoamine transporters between duloxetine and venlafaxine are in support of a series of other studies [4, 20, 26, 33, 34]. This discrepancy has been also replicated in a recent study [38], although there has been some discrepancies in affinity results possible due to laboratory techniques and other factors. In the study, the affinity and selectivity of milnacipran, duloxetine, and venlafaxine were compared for the human serotonin, norepinephrine, and dopamine transporters [38]. Study results showed that milnacipran was the most norepinephrine selective compound in comparison with duloxetine and venlafaxine [38]. Duloxetine was found to be the most potent of the agents tested in blocking the reuptake of serotonin. Venlafaxine, in contrast, selectively bound to the serotonin transporter, but not the norepinephrine transporter [38]. Milnacipran established high affinity to both serotonin and norepinephrine transporters as well as showing a strong ability of uptake inhibition at both serotonin and norepinephrine transporters [38]. The potency of affinity and reuptake inhibition between three SNRIs for the transporters are presented in Table **1**.

**Fig. (1):**
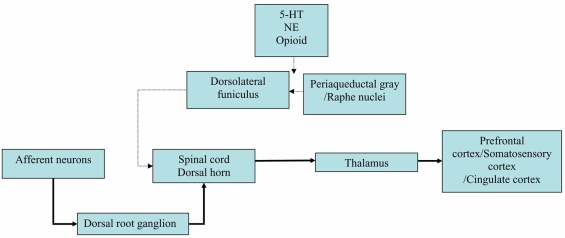
Circuit of pain modulatory pathway. Abbreviations: 5-HT, serotonin; NE, norepinephrine. Thick arrow indicates ascending pain pathway and thin arrow represents descending inhibitory pain pathway.

In this context, it is intriguing that the net effect of SNRIs results in increment of extracellular 5-HT and NE levels in prefrontal cortex, which is correlated with uptake blockade increasing extracellular levels of the neurotransmitters in the synapse [5]. A number of experimental studies on chronic pain have consistently shown its engagement with prefrontal cortex activity [41, 42]. Cognitive modulations of pain are related to activation of regions of interest in several prefrontal brain areas (dorsolateral prefrontal cortex, ventrolateral prefrontal cortex and anterior cingulated cortex), where eventually modulate the central and peripheral pain pathways in some crucial regions in the CNS and spinal cord (i.e., thalamus, periaqueductal gray and dorsal horn, see Fig. **1**) [41, 42]. In fact the dorsolateral prefrontal cortex is directly and indirectly connected to the anterior cingulate cortex and thalamus, and finally to the periaqueductal gray, a critical part of the descending pain modulatory system [42]. In addition, the antidepressant effect has been shown to be involved with activation of such brain areas [10]. Hence future researches should integrate in proper manner the findings of experimental model for binding affinity and uptake inhibition to monoamine transporter and the data of brain activations in functional imaging studies using with SNRIs in patients with pain.

## CLINICAL STUDIES

TCAs that inhibit the reuptake of norepinephrine or both norepinephrine and serotonin, such as amitriptyline and desipramine, have demonstrated efficacy in the treatment of chronic pain conditions such as diabetic neuropathy, fibromyalgia, chronic headaches, and post-herpetic neuralgia [30]. Their ability to relieve pain in these conditions appears to be independent of their antidepressant effect and may be directly related to their effect on neuronal reuptake of serotonin and norepinephrine and in part by the increased duration or concentration of serotonin and norepinephrine in synapses associated with central pain integration [25]. 

There has been extensive research to isolate the specific involvement of each neurotransmitter in the pain pathways. SNRIs have been shown to be more efficacious than monoamine oxidase inhibitors in producing analgesia, with the analgesic effects of the antidepressants beginning before the antidepressant effects. It is known that antidepressants are pharmacologically similar to opiates. The above observations combined, indicate that serotonin and norepinephrine do have a significant role to play in pain control [30].

A meta-analysis of 39 placebo controlled studies was done to estimate the effect of antidepressant-induced analgesia in chronic non-malignant pain. The average chronic pain patient who received an antidepressant treatment had less pain than 74% of the chronic pain patients who received a placebo, which translates to a statistically significant pain decrement and a statistically significant difference between drug and placebo [30]. A recent meta-analysis of trials of antidepressants in the treatment of fibromyalgia indicates that TCAs have superior efficacy compared to placebo [27].

Duloxetine has demonstrated efficacy in the treatment of fibromyalgia in two similarly designed pivotal randomized, double-blind, placebo-controlled clinical trials (RCTs) [1, 3], and it has become the first antidepressant medication to obtain US FDA approval for this indication. A pooled data analysis from these two RCTs provides greater statistical power and more clearly illustrates the efficacy of duloxetine for pain, functional impairment, and quality of life in fibromyalgia patients [2]. The pooled data set included patients treated with duloxetine 60 mg QD or 60 mg BID (n = 326) *vs*. placebo (n = 212). Duloxetine demonstrated significantly greater improvement in the two principal efficacy measures of Brief Pain Inventory (BPI)-average pain severity score and the Fibromyalgia Impact Questionnaire (FIQ) total score as early as week 1 and continuing through the study endpoint (week 12). Duloxetine also established superiority to placebo on measures of quality of life and functional outcome. The proven acute efficacy of duloxetine for fibromyalgia was replicated in a 6-month RCT which also indicated the durability of treatment response [35]. Doses studies included 60 mg/day and 120 mg/day, with the 60 mg/day dose showing better tolerability.

Another SNRI with demonstrated efficacy in the treatment of fibromyalgia is milnacipran, which is officially approved for the treatment of major depressive disorder in European and Asian countries, but not by the US FDA [31]. A New Drug Application (NDA) was submitted in December of 2007 to the US FDA for milnacipran in the treatment of fibromyalgia, which includes data from phase III trials involving more than 2,000 patients [31]. The potential efficacy of milnacipran in the treatment of fibromyalgia has been reported in two published flexible dose, 12-week RCTs [13, 39], and three unpublished fixed dose, 15-week [7], 6-month [8] and serial 1-year [14] RCTs. In such studies, milnacipran was effective in pain and functional domains of fibromyalgia. Currently existing acute and maintenance treatment data suggests that doses of 100 mg/d and 200 mg/d may be equally efficacioust, with better tolerability shown for the 100 mg/d dose of milnacipran.

A more limited body of data supports the efficacy of the SNRI venlafaxine in the treatment of fibromyalgia. A small short RCT (n=90) of venlafaxine 75 mg/d was inconclusive [45], although a subsequent extended RCT (n=15) for 12 weeks showed that venlafaxine treatment was associated with significant improvement in mean pain intensity and in disability compared to placebo; the small sample size limits definitive conclusions from this study [36].

Of note, all three of these SNRIs have demonstrated efficacy in placebo-controlled studies in other pain syndromes, including neuropathic pain [32, 40, 44], headache [46], and multiple somatic pains [21].

## ISSUES TO BE ADDRESSED IN THE FUTURE

There have been no direct comparative trials between SNRIs and other antidepressants (e.g., SSRIs and TCAs) as well as other agents (e.g., gabapentin, pregabalin, sodium oxybate, and pramipexole) that showed potential effectiveness for pain control. With these trials clinicians may have more valuable information about the differential role among each agent and hence a benefit/risk ratio could be taken into consideration on the choice of proper medication for individual patient. Although the comparative advantage of an SNRI compared to a TCA or a medication like gabapentin could be definitely proved by a direct comparison trial, it should be unlikely to be conducted due to several issues at this point, i.e., less likelihood of financial support from pharmaceutical company, different doses between agents for pain control, comedications, multiple and complicated etiopathogenesis of pain (i.e., combined psychological and physical factors).

Although meta-analysis only reveals indirect comparisons about relative impact of independent variables, strength of relationship between variables and overall effectiveness of interventions among therapeutic agents and has publication bias, meta-analytic findings are consistently report the direct analgesic effects of antidepressants having a property of serotonin and norepinephrine reuptake inhibition such as TCAs and SNRIs [12]. In such report, antidepressants including SNRIs were also found to improve health-related quality of life (standardized mean differences (SMD), -0.31; 95% CI, -0.42 to -0.20) as well as proving analgesic effects (SMD, -0.43; 95% confidence interval [CI], -0.55 to -0.30)[16]. Hence larger and more well-designed meta-analytic data may also provide useful information on this research field.

Clinical studies with different clinical domains, different age-gender groups, those with comorbidity and refractory cases will also provide further information about the role of SNRIs for such conditions.

## CONCLUSION

Copious data support a role for antidepressants in the treatment of chronic pain syndromes, particularly neuropathic pain and fibromyalgia. The mechanisms underlying this role include inhibition of serotonin and norepinephrine reuptake leading to enhanced descending inhibition of centrally sensitized pain. Antidepressants have demonstrated analgesic effects in clinical studies in humans and in animal models of pain. This body of data suggests greater effects with dual-acting antidepressants enhancing serotonergic and noradrenergic transmission such as TCAs and SNRIs, with evidence also existing for a smaller effect from medications enhancing norepinephrine alone. Data supporting pain-reducing effects of selectively serotonergic medications is more limited, presumably due to the role of serotonin in both inhibiting and enhancing pain *via* descending pathways. As physiological mechanisms modulating pain expression are further clarified, improved pharmacological interventions for central pain syndromes is expected.

## Figures and Tables

**Table 1.** The Potency of Affinity (Ki, nmol/L) and Reuptake Inhibition Between Three SNRIs for the Transporters*

	Transporter Binding		Reuptake Inhibition	
	Serotonin	Norepinephrine	Serotonin	Norepinephrine
Duloxetine	0.07±0.01	1.17±0.11	3.7±1.1	20±6
Milnacipran	8.44±1.57	22±2.58	151±24	68±10
Venlafaxine	7.8±0.28	1,920±158	145±18	1,420±240

* Results have discrepancy between studies and this table was only modified from one study since it has simultaneously compared the potency of affinity and uptake inhibition between duloxetine, milnacipran and venlafaxine [38].
